# The Relationship Between Occupational Demands and Well-Being of Performing Artists: A Systematic Review

**DOI:** 10.3389/fpsyg.2019.00393

**Published:** 2019-03-04

**Authors:** Simone Willis, Rich Neil, Mikel Charles Mellick, David Wasley

**Affiliations:** Cardiff School of Sport and Health Sciences, Cardiff Metropolitan University, Cardiff, United Kingdom

**Keywords:** occupational stress, well-being, wellbeing, happiness, performing artists, musician, dancer, systematic review

## Abstract

**Background:** Performing artists are exposed to a range of occupational demands from organisational, interpersonal and intrapersonal sources, which may impact their well-being. The aim of this systematic review was to evaluate and synthesise the literature where researchers have considered the relationship between occupational demands and well-being in performing artists.

**Methods:** A mixed-methods systematic review was conducted including professional and student performing artists. Quantitative, qualitative and mixed-methods study designs were eligible for inclusion in the review. A total of 14 databases were searched from their inception through to October 2017, including MEDLINE, EMBASE, and Scopus. Critical appraisal was conducted using the Mixed-Methods Appraisal Tool and results presented as a narrative synthesis.

**Results:** A total of 20 studies were included in the review, comprising of quantitative (*n* = 7), qualitative (*n* = 9) and mixed-methods (*n* = 4) study designs. Several frameworks of occupational stress and well-being were explored in relation to the results. Organisational, social and emotional demands were associated with lower well-being. Conversely, music-making, performance activities and social support were reported to be resources and were related to higher well-being.

**Conclusion:** This systematic review highlights the need for researchers in this field to adopt methodologically robust study designs, which are informed by appropriate theoretical frameworks. The paucity of high quality and theoretically informed research in this area is a hindrance to the development of evidence-based interventions for this population.

## Introduction

Performing artists span a range of disciplines and performance environments and are required to possess a diverse skill set to develop and maintain successful careers. They are expected to display technical mastery, portray expressive qualities, acquire business acumen, and interact with the public and other stakeholders (Williamon, 2004; Vaag et al., 2014). Research on musicians and dancers report similarities in the environment experienced in their careers: both often carry out a range of roles including performing, teaching, and working in a self-employed capacity (Bennett, 2009). Additionally, performing artists such as actors, comedians, and circus artists work in similar environments and often hold multiple occupational roles (Throsby and Zednik, 2011). Whilst some performing artists may be affiliated to a particular organisation, many work in a freelance capacity holding concurrent contracts (Mills, 2004).

This multifaceted professional identity exposes performing artists to a variety of occupational demands categorised under organisational, interpersonal, and intrapersonal domains. Occupational demands refer to aspects of the working environment that may impact an individual either physically or psychologically. A systematic review of the literature that focused only on the occupational demands experienced by musicians identified seven categories: public exposure, personal hazards, repertoire, competition, job context, injury and illness, and criticism (Vervainioti and Alexopoulos, 2015). Equally, dancers experienced pressure to conform to a prescribed body type, endure heavy rehearsal schedules and were required to navigate multiple interpersonal relationships within their respective dance company (Noh et al., 2009).

The impact of physical demands on the physical health of performing artists has received significant attention in the literature and been explored in musicians (e.g., Zaza, 1998; Williamon and Thompson, 2006; Kok et al., 2016; Gembris et al., 2018), dancers (Jacobs et al., 2012; Kenny et al., 2016b), and circus artists (Shrier et al., 2009; Wolfenden and Angioi, 2017). Playing-related musculoskeletal disorders have been evidenced to have significant implications for performing artists such as impacting on performance quality and leading to absence from work (Ackermann et al., 2012). In addition to the physical demand of playing an instrument, Rickert et al. (2013) found that organisational demands such as heavy schedules and technically demanding repertoire were associated with increased risk for injury in orchestral musicians.

Performing artists are also exposed to a number of interpersonal demands and are required to maintain a multiplicity of relationships, including those with colleagues, peers, management and audiences, who may have different and conflicting agendas. Researchers have reported that positive interpersonal relationships with colleagues are necessary for a harmonious working environment and also affect the quality of the artistic product (Lim, 2014; Dobson and Gaunt, 2015). Interpersonal skills are particularly tested when performing artists are transitioning into the profession and seeking new employment opportunities, particularly when in a freelance capacity (Dobson, 2010a). This is due to the need to work within an already established team and respond to interpersonal cues (Dobson and Gaunt, 2015). Additionally, this period may be accompanied by demands such as financial insecurity and competition with peers (Creech et al., 2008). The transition into the profession is often not linear and may occur whilst individuals are enrolled on performing arts awards at higher education institutions, meaning they must cope with both educational and professional demands concurrently.

Intrapersonal demands, such as perfectionistic strivings, may also impact on performing artists through the occupational setting (Kenny et al., 2004). Within music, intrapersonal demands have frequently been explored in relation to music performance anxiety (MPA; e.g., Kenny, 2011). In a study by Pecen et al. (2018), professional musicians recognised experiencing psychological challenges related to coping with affective experiences connected with expressive performance and managing personal expectations. Further, research with dancers (Mainwaring and Finney, 2017) and circus artists (Shrier and Hallé, 2011) has highlighted the potential negative effect of intrapersonal demands and their relation to injury.

Research from the occupational stress literature with university staff (Mark and Smith, 2012a) and nurses (Mark and Smith, 2012b) suggests that occupational demands may negatively impact on both cognitive and affective well-being outcomes (Lazarus, 1999). Whilst some facets of the occupational environment of performing artists may have a negative impact on well-being, other aspects may provide enabling conditions which facilitate well-being. Many performing artists are self-employed and research with creative professionals suggests that self-employed workers have greater opportunities for autonomy, creativity and learning experiences (Bujacz et al., 2017). Within the literature, self-employment has been found to relate to higher job satisfaction (Andersson, 2008; Warr, 2018) and life satisfaction (Binder and Coad, 2016) when compared to employment. However, self-employment has also been found to relate to short-term psychological distress (Reid et al., 2018) and individuals may experience job insecurity, which may have a negative impact on well-being (de Witte et al., 2016).

### Conceptualisations of Occupational Stress

Within the literature on performing artists, researchers have drawn on a range of conceptual frameworks to inform their explorations into occupational demands and well-being outcomes. These include the job demand-control(-support) model (Karasek, 1979; Johnson and Hall, 1988), the effort-reward imbalance model (Siegrist, 1996), the job demand-resources model (Bakker and Demerouti, 2014) and psychosocial models of occupational stress. This section explores research in this area on performing artists alongside prominent frameworks in the occupational stress and well-being literature.

Research, which draws on the job demand-control model (JDC; Karasek, 1979), found that musicians experience demands such as irregular working hours, repetitive work and competition amongst colleagues (Steptoe, 1989). In a study with conservatoire musicians, Akel and Düger (2007) found that older students experienced higher levels of psychological job demands and greater job insecurity. However, the authors do not report on the specific job demands that musicians faced and further studies which test associations between the variables of the JDC model are required. The JDC model (Karasek, 1979) suggests an interaction effect, proposing that individuals exposed to high job demands and low job control may experience a negative impact on well-being. Within the model, job control is composed of two dimensions: decision authority, which relates to the control an individual has over their work, and skill discretion, which refers to the variety of skills an employee uses (Mark and Smith, 2008). This model has been developed to include social support as a potential moderator of the relationship between occupational demands and outcomes of the stress process (Johnson and Hall, 1988). Within the demand-control-support model (JDCS), Johnson and Hall (1988) found that individuals who had lower support from co-workers experienced higher levels of occupational stress. These models have had a significant impact on the occupational stress literature with numerous studies assessing the relationships between the dimensions (Häusser et al., 2010).

Whilst rarely applied in the performance artist literature, the effort-reward imbalance model (ERI; Siegrist, 1996) has also informed much of the occupational stress literature and suggests a reciprocal relationship between effort and reward. Effort relates to both extrinsic factors, such as occupational demands, and intrinsic factors, such as motivation. The reward dimension comprises the components salary, esteem, career opportunities and job security (Peter and Siegrist, 1999). Exposure to situations which comprise high effort and low reward may have a negative impact on well-being (van Vegchel et al., 2002).

A criticism of the JDCS and ERI models is the lack of inclusion of the individual in the stress process. Lazarus' cognitive-motivational-relational model is a transactional approach and considers the role of individual appraisal in the stress process (Lazarus, 1999). Lazarus suggested that there are two stages of appraisal: primary and secondary. Primary appraisal involves the individual assessing whether a potential demand has implications for their personal goals and values. Where there are implications for the individual, the demand will be evaluated in terms of harm, threat, loss or challenge. This leads to secondary appraisal, which is the assessment of coping options (Lazarus, 1999). This model represents the complexities of the stress process; however, there are difficulties in applying it to empirical research due to the complexity of assessing individual appraisal.

Another influential framework within the occupational stress literature is the job demands-resources model (Bakker and Demerouti, 2014). The authors suggested that occupational stress models should allow for the incorporation of the most salient job demands dependent on the specific occupation under study and a wider range of factors, such as emotional demands and performance feedback, should be considered (Bakker and Demerouti, 2007). Aspects of the occupational environment are categorised as either job demands or job resources. Job demands can be defined as “physical, social, or organizational aspects of the job that require sustained physical or mental effort [to manage]” (Demerouti et al., 2001, 501). Job resources are aspects of an occupation that may support the completion of work tasks, reduce occupational demands and any related physical or psychological outcome and/or facilitate the personal development of employees (Schaufeli and Bakker, 2004). Considering the literature on performing artists, Vaag et al. (2014) used the JDR model to guide qualitative research with freelance musicians. This research found that both demands and resources were important aspects of the occupational environment for freelance musicians, who highlighted the importance of social support from their personal and professional networks alongside personal resources such as developing resilience and maintaining a passion for music.

### Conceptualisations of Well-Being

Several studies have examined well-being outcomes in the workplace of performing artists drawing on frameworks such as self-determination theory (Ryan and Deci, 2001), hedonic well-being (Diener et al., 1999) and eudaimonic well-being (Ryff, 2014). Considering the impact of occupational demands on the well-being of professionals in artistic roles, Tuisku et al. (2016) found that employment type (e.g., full-time) and stability were related to well-being outcomes: individuals who were in full-time stable roles reported higher levels of cognitive and affective well-being compared to those with irregular working hours. This study conceptualised well-being holistically as including hedonic, eudaimonic and social well-being dimensions (Fisher, 2014). Within the literature, a consensus on the definition of well-being has not yet been reached. The difficulties of defining well-being are discussed by Dodge et al. (2012) and within the psychology literature well-being has traditionally been defined from two perspectives: hedonic and eudaimonic (Waterman, 2008; Biswas-Diener et al., 2009).

Hedonic well-being, sometimes called subjective well-being, encompasses affective and cognitive dimensions (Diener et al., 1999). The affective dimensions of hedonic well-being are positive and negative affect, which are measured independently, and the cognitive dimension is life satisfaction. Early research on emotions found that positive and negative emotions were not opposite ends of a continuum but rather independent dimensions (Bradburn and Caplovitz, 1965; Bradburn, 1969). Whilst these dimensions have shown moderate inverse correlation, they demonstrate distinct constructs (Diener et al., 1995). This led to the inclusion of two affective dimensions within hedonic well-being: positive affect and negative affect. The third dimension of subjective well-being, life satisfaction, is considered to be a global evaluative judgement of an individual's well-being, which demonstrates independence from the affective dimensions (Lucas et al., 1996). Assessing both the cognitive and affective dimensions of hedonic well-being allows for a holistic evaluation of subjective well-being (Diener and Seligman, 2004), as individuals may include judgements not related to affective states when reporting life satisfaction. For example, individuals may include areas such as success when reporting life satisfaction, which may not be reflected in reports of affect (Pavot and Diener, 2008).

The hedonic conceptualisation of well-being is centred on the subjective perspective of the individual, allowing individuals rather than researchers to decide on the factors that contribute to their well-being (Diener et al., 1998). Criticisms of the hedonic approach include the lack of theory-based research guiding the conceptualisation and the omission of important aspects of experiencing a fulfilling life (Ryff, 1989). In an attempt to address this, Ryff developed a six-factor model of well-being, referred to as eudaimonic well-being (Ryff, 1989), which features objective dimensions of well-being. The term eudaimonia has its roots in Aristotle's consideration of a virtuous life and has been translated as “happiness,” “fulfilment,” and “flourishing” (Brown, 2009). In accord with this, eudaimonic well-being takes a wider perspective and researchers in this tradition are concerned with the fulfilment of human potential and the flourishing of the individual. Ryff (2014) suggests that eudaimonic well-being is made up of six factors: self-acceptance, positive relations with others, autonomy, environmental mastery, purpose in life and personal growth. These six dimensions represent cognitive evaluations on aspects of an individual's life.

The distinction between hedonic and eudaimonic well-being has been questioned in the literature, with researchers suggesting theoretical similarities in conceptualisations (Kashdan et al., 2008) and the potential for the perspectives to complement each other (Waterman, 2008; Huta and Waterman, 2014). For instance, Kashdan et al. (2008) suggest similarities in the life satisfaction dimension of subjective well-being and the purpose in life dimension of eudaimonic well-being. Additionally, empirical research suggests correlations between dimensions of hedonic and eudaimonic well-being (Keyes et al., 2002).

Within the occupational literature, well-being has been operationalised in a variety of ways. Considering hedonic well-being, life satisfaction is frequently operationalised as job satisfaction (Fisher, 2010). One example of this within the performing arts is a study by Cahalan and O'Sullivan (2013), who found that Irish dancers reported a high level of job satisfaction citing reasons such as opportunity to travel and being remunerated for a career they were passionate about. The disagreements on the conceptualisation of well-being have led to a plethora of further operationalisations, which include engagement, organisational commitment, momentary affect and vigour (Fisher, 2010). The operationalisation of well-being for the purpose of this systematic review incorporates both hedonic and eudaimonic well-being domains; psychological functioning of the individual represented by only cognitive evaluations relating to the quality of life, or cognitive evaluations and affective outcomes combined relating to the quality of life. In other words, to meet the inclusion criteria for the review, articles must use a holistic operationalisation of hedonic well-being or a dimension of eudaimonic well-being.

### Rationale

Whilst an extensive body of literature over the past 30 years has been developed around the impact of occupational demands, the focus has been on outcomes such as health, injury and MPA. Lewchuk (2017) suggested that negative affect experienced by individuals due to precarious employment had an adverse impact on their relationships inside and outside the occupational environment. Given the nature of work carried out by performing artists, organisational, interpersonal, and intrapersonal occupational demands such as these may negatively impact on their well-being. Synthesising the literature on this topic will allow for an unbiased evaluation and, as a result, identification of a direction for future research, which will enable the development of evidence-based interventions to support performing artists.

### Aim and Objectives

The aim of this systematic review was to evaluate and synthesise the literature that has focused on the relationship between occupational demands and well-being in performing artists. The objectives of this systematic review were to critically appraise the quality of the literature, synthesise the findings of previous research on this topic and identify future research foci on the well-being of performing artists, particularly that of musicians and dancers, in order to provide an explicit foundation for evidence-based support programmes and interventions.

## Methods

### Study Design

A mixed-methods systematic review was chosen to assess the full extent of literature on the topic. Mixed-methods systematic reviews integrate the results of primary research studies that use quantitative, qualitative or mixed-methods approaches (Sandelowski et al., 2012). This allows for the best use of the available research to create evidence summaries which are able to inform decision-makers about appropriate interventions and directions for future research (Pearson et al., 2014). The integration of different research methods allows for the synthesis of research which is able to provide statistically meaningful results with insight into the experiences of those concerned.

The inclusion criteria was developed using the SPIDER search tool (Cooke et al., 2012). The SPIDER tool is a method for defining the sample, phenomenon of interest, research design, evaluation, and research type (methodology) to be studied. Defining these parameters is relevant for identifying research which is quantitative, qualitative, and mixed-methods in design, meaning the tool is applicable for conducting a mixed-methods systematic review. Specifically, identifying the areas of phenomenon of interest and evaluation are appropriate for undertaking a systematic review with a broad question, which does not seek to evaluate the effectiveness of interventions. The systematic review was conducted following guidance from the Preferred Reporting Items for Systematic Reviews and Meta-Analyses (PRISMA) Statement (Moher et al., 2009), which provides guidance on the process of conducting a systematic review and appropriate reporting standards (Liberati et al., 2009).

Critical appraisal of study quality was conducted using the Mixed-Methods Appraisal Tool [MMAT; Pluye et al., 2011], which is designed to facilitate concurrent critical appraisal of quantitative, qualitative and mixed-methods primary research in mixed-method systematic reviews (Pace et al., 2012). The ability to critically appraise all research designs with one tool facilitates a standardised approach which allows comparison to be made between studies of different methodologies (Crowe and Sheppard, 2011). Additionally, the MMAT shows good reliability and efficiency when used independently by multiple reviewers (Souto et al., 2015) and has been used in systematic reviews on a wide range of topics (Hong et al., 2018). The MMAT allows for the use of a different set of criteria for the appraisal of five different study designs: qualitative, randomised control trials, non-randomised quantitative, observational descriptive, and mixed-methods (Pace et al., 2012). Further details of the MMAT criteria for qualitative, observational descriptive, and mixed-methods studies is provided, as these were used in this systematic review. The qualitative criteria used in the MMAT includes four areas: (1) appropriateness of participants and sampling procedure; (2) data analysis process including method of data collection, data format and data analysis; (3) consideration of the influence of setting for data collection; and (4) consideration of the influence of the researchers' prior ontological and epistemological beliefs. Criteria for assessing observational descriptive studies includes: (1) sample source and size; (2) whether the sample was representative of the population (3) suitability of the measures; and (4) response rate. The critical appraisal of mixed-methods included the qualitative and quantitative descriptive criteria above along with criteria specific for mixed-methods studies. These included the following three areas: (1) relevance of mixed-methods design; (2) synthesis of data; and (3) consideration of limitations of the methodology.

As the review included a range of study designs, a narrative synthesis approach was considered suitable for reporting the results. Narrative synthesis is appropriate for integrating research from diverse methodologies due to the possibility of considering a variety of research designs in juxtaposition (Dixon-Woods et al., 2005). This systematic review used an integrated design for the analysis and synthesis of included data, meaning that data for all studies was pooled and analysed concurrently (Sandelowski et al., 2006). Integrated designs for systematic reviews can be considered applicable when the analysis of studies using different methodologies are interpreted in consideration of the same research question and the data can be meaningfully presented in the same way (Sandelowski et al., 2006). In this instance, a preliminary synthesis was developed form the extracted data by clustering data, vote counting and tabulation, whilst conceptual ideas webbing was used to abstract data into higher order concepts (Popay et al., 2006).

### Participants, Exposure, Outcome

The search strategy included professional performing artists in the fields of music, dance, acting, circus performance and comedy. Due to the crossover between student and professional status, it was deemed appropriate to include both professional performing artists and individuals studying arts awards at educational institutions in this systematic review. The phenomenon of interest explored in the systematic review was the relationship between occupational demands and well-being. The inclusion criteria encompassed all study designs in order to capture the full range of literature on the topic to date. Studies were included where the impact of occupational demands on well-being was explored and well-being was operationalised as above. Where studies adopted a partial operationalisation of hedonic well-being and measured only affective outcomes (e.g., positive affect) they were not included.

### Systematic Review Protocol

No protocol previously existed for the conduct of a systematic review on the topic. Therefore, a protocol was developed in line with Moher et al. (2015), which included the review question, search strategy, inclusion and exclusion criteria, and details of the choice of tool for quality assessment. The protocol also included details on the process for the production of a narrative synthesis. A copy of the protocol is available from the lead author on request.

### Search Strategy

An electronic search strategy was employed using the following databases: (i) EBSCOhost (including Art Full Text, SPORTDiscus, EBSCOhost, Education Research Complete, GREENfile, Hospitality and Tourism Complete, Library, Information Science and Technology Abstracts, MEDLINE, Regional Business News, Business Source Premier); (ii) OvidSP [including PsycArticles, PsycINFO, EMBASE, and MEDLINE (including MEDLINE ePub and In-Process)]; (iii) Scopus. These databases were selected due to their relevance to the topic, which would ensure that all appropriate material was found. The following keywords were included in the search strategy; *musician, artist, dancer, “performing art,” well-being, wellbeing*, and *satisfaction*. Inverted commas were used around the term “performing art” to ensure searches returned articles related to performing art as opposed to returning articles related to performing and art. Boolean logic operators and truncation were used to combine keywords in the search strategy for each database. When conducting the search, filter boxes were used for “peer-reviewed” articles and “English language.” No date filters were used in the search strategy. In addition to an electronic search, the two journals Medical Problems of Performing Artists and Psychology of Music were handsearched. These journals were selected due to their contextual relevance and to ensure the completeness of the search strategy (Hopewell et al., 2007). Once appropriate studies were identified for inclusion, the reference lists of each study were checked for any additional studies that were relevant to the systematic review.

### Data Sources, Study Selection, and Data Extraction

All databases were searched from their inception until the date of the final search (i.e., 13 October 2017). Where handsearches were conducted, these were also carried out from the journals' first published issue.

The following inclusion criteria was applied for articles to be included in the systematic review: (i) peer-reviewed journal articles; (ii) articles published in the English language until the date searched (i.e., 13 October 2017); (iii) articles that focused on professional performing artists, or focused on performing artists studying performing arts awards in educational settings; (iv) articles included adults aged 18 years old or above as participants; (v) articles that measured the relationship between occupational demands and well-being. The following exclusion criteria was applied to articles to ensure that only relevant articles were retained for the systematic review: (i) non-peer-reviewed journal articles; (ii) articles that did not include professional performing artists or performing artists studying for performing arts awards in educational settings; (iii) articles that included children or individuals under the age of 18 years old; (iv) articles that did not assess the relationship between occupational demands and well-being; (v) editorials and forewords; (vi) book chapters, book reviews and book synopses; (vii) conference proceedings and conference abstracts; (viii) unpublished theses. See Appendix A for full details of inclusion and exclusion criteria. Where an article included the eligible population as a subset, they were included if the data for the subset could be extracted from the main data set.

In accordance with the PRISMA statement (Moher et al., 2009) citations were screened and duplicates removed. Following this, titles and abstracts were screened against the inclusion and exclusion criteria. Remaining articles were screened at full-text against the inclusion and exclusion criteria. This process was conducted by the first author (SW) and checked by the fourth author (DW). All authors discussed and agreed on the final list of studies to be included in the review.

A data extraction form (Appendix B) was created and the first (SW) and fourth (DW) authors achieved consensus on the data to be extracted. The data extraction form was piloted on a subset of four studies (representing 20% of the included studies). Data extracted included: reason for inclusion in the review, author(s), year of publication, study location, participant characteristics (e.g., occupation, age), context (e.g., symphony orchestra, conservatoire), sampling method, response rate, aims, study design, conceptual framework, variables, themes explored, outcome measures, validity or credibility, method of analysis, results summary, author identified limitations, additional limitations, implications for future research, and funding body or sponsor. Data extraction was completed by the first author (SW) and checked by the second author (RN).

### Data Analysis

The Mixed Methods Appraisal Tool [MMAT; (Pluye et al., 2011)] was used to assess the methodological quality of studies in the systematic review. This tool was selected as it can be used with quantitative, qualitative and mixed-methods studies, and to ensure standardisation of assessment across studies. The validity and reliability of the MMAT have been assessed (Pace et al., 2012; Souto et al., 2015) and the tool found to be appropriate for the appraisal of studies in mixed-method systematic reviews. Quality appraisal of a subset of four studies (representing 20% of those included) was conducted by the first and fourth authors independently (SW and DW). Inter-rater reliability was calculated by means of Cohen's kappa (Cohen, 1960) using the software package SPSS (V. 23.0.0.3) and found to be 0.736, which represents substantial agreement (Landis and Koch, 1977). Disagreements on study quality were resolved by discussion between the first and fourth authors. Following this, quality assessment using the MMAT was conducted by the first author for the remaining studies.

Due to the diverse range of methodologies and heterogeneity in the included studies, a narrative synthesis was deemed appropriate for presenting the results of the systematic review (Dixon-Woods et al., 2005). Guidance from Popay et al. (2006) was followed on the development of narrative synthesis and extracted data were visualised using tabulation, clustering and vote counting. Textual descriptions and ideas webbing were used to explore relationships between occupational demands and cognitive and affective well-being domains. Themes for the discussion were developed deductively considering conceptualisations of both occupational stress and well-being. Dimensions of the job demands-control(-support) model (Karasek, 1979; Johnson and Hall, 1988), effort-reward imbalance model (Siegrist, 1996) and the job demands-resources model (Bakker and Demerouti, 2014) were considered alongside those of hedonic (Diener et al., 1999) and eudaimonic (Ryff, 2014) well-being frameworks.

## Results

### Study Selection and Characteristics

A total of 336 articles were identified from database searches, handsearches, and reference list checking. After 106 duplicates were removed, 230 titles and abstracts were screened against the inclusion and exclusion criteria. This led to the exclusion of 152 articles. The full-text of the 78 articles which remained were obtained and screened for eligibility, which led to the exclusion of a further 58 articles. All articles excluded at full-text are listed in Appendix C alongside reasons for exclusion. In total, 20 articles were retained for inclusion in the systematic review and a summary is presented in Table 1.

**Table 1:** Summary of included studies.

**References**	**Context**	**Number of participants**	**Participant characteristics**	**Study design**	**Aim(s)**	**Variables/Themes**	**Results**
Abeles and Hafeli, 2014	Symphony orchestra musicians	47	USA F16 M31	Qualitative: Semi-structured interview	Explore motivations of musicians to contribute to school education programmes and assess how such participation affects career perceptions	Motivations for participation Programme experiences	Delivering the programme was experienced as an opportunity for professional development and led to positive relationships with the community, autonomy, self-expression and positive affect.
Allmendinger et al., 1996	Symphony orchestra musicians	1,123	UK, USA, Germany	Mixed-methods: Interview Observation Questionnaire Archival documents	Explore differences in orchestras and musicians' career profiles from the UK, USA and Germany	Operational information Orchestra integrity Player involvement Resources Player recognition Recruitment procedures Satisfaction Career mobility Gender representation Perceptions on gender representation	Musicians were satisfied with relationships with colleagues, though scored low for satisfaction with pay and management.
Ascenso et al., 2017	Classical musicians	6	Germany, Portugal, Spain, UK F3 M3 Age range 32–52 (mean = 43.17)	Qualitative: Interview Diary	Understand the well-being of professional musicians	Developed from PERMA profiler	Musicians had high well-being. Factors contributing to well-being included understanding identity, making music and relationships. Challenges to well-being included relationships with management, monotony in rehearsals and transition into the profession.
Bodner and Bensimon, 2008	Band musicians	38	Israel F8 M30 Age range 22–45 (mean = 28.82)	Quantitative; 2 × 2 (condition × time) mixed model MANOVA	Assess the adjustment of solo singers after performance and explore mental health	Affect Self-esteem Purpose in life Mental Health	Singers scored higher on purpose in life, negative affect and positive affect before performance compared to after. Higher well-being and lower distress were related to higher purpose in life after performance.
Brodsky, 2006	Symphony orchestra musicians	54	UK Age range 22–55 (mean = 35.5)	Qualitative: Semi-structured interview	Explore the occupational experiences of orchestral musicians	Gains, risks and costs of orchestral career	Factors contributing to well-being included relationships with colleagues, emotional satisfaction, sharing performances, task variety, learning, feelings of accomplishment. Challenges to well-being included maintaining relationships, cognitive effort required for performance, maladaptive coping, low autonomy and limited career progression.
Burgoyne et al., 1999	University student actors	15	USA	Qualitative: Interview	Understand the impact of acting on student actors	N/A (Grounded theory approach)	Contributors to well-being included development of empathetic and relationship skills and experiencing meaning. Challenges to well-being included relationships with directors, distressing content and maintaining personality characteristics.
Cooper et al., 1989	Popular musicians	70	UK M70 Age range 22–62 (mean = 40)	Mixed-method: In-depth interview Questionnaire	Assess the major sources of stress experienced by popular musicians	Stress Personality	Low job satisfaction was related to working with groups that lacked personal and professional cohesion.
Dobson, 2010b	Classical and jazz musicians	18	UK F7 M11 Age range 21–34 (mean = 24.6)	Qualitative: Semi-structured interview	Explore the occupational demands placed on classical and jazz musicians and explore differences in experiences of autonomy	Creativity Work Control Demands Lifestyle Well-being	Identity, emotional investment and autonomy related to well-being. Musicians highly identified with their profession and experienced guilt after mistakes. Jazz players experienced greater autonomy compared to orchestral musicians.
Draugelis et al., 2014	University dance students	182	USA F157 M25 Age range 18–43 (mean = 20.4)	Quantitative: Cross-sectional questionnaire	Assess the contributions of motivational climate, dance performance anxiety and dance self-concept to well-being	Motivational Climate Dance self-Concept Dance Anxiety Well-being	Motivational climate and dance self-concept significantly related to well-being of dancers.
Johansson and Theorell, 2003	Orchestra musicians	250	Sweden F93 M155 Mean age = 39	Quantitative: Cross-sectional questionnaire	Identify factors determining well-functioning groups and issues for orchestral musicians	Orchestra status Job security Quality of work tasks Psychosocial factors Health	Quality of work tasks, psychosocial factors and social support significantly correlated with well-being. Musicians in elite orchestras and those with lower support reported lower well-being.
Kenny et al., 2016a	Orchestra musicians	Survey *n =* 380 Physical examination *n =* 407	Australia F206 M198 Mean age = 42.1	Quantitative: Cross-sectional questionnaire Physical examination	Explore factors impacting on health	Performance-related musculoskeletal pain disorders (PRMD) Music performance anxiety Practice and organisational factors Prevalence of bullying Occupational satisfaction	Job satisfaction was consistent across orchestra types. Musicians in stage orchestras were more satisfied with their workplace, employers, relationships with management, colleagues, pay and career progression.
Kivimaki and Jokinen, 1994	Orchestral musicians	93	Finland F28 M65	Quantitative: Cross-sectional questionnaire	Assess job perceptions and well-being among musicians and compare results to other occupational groups	Job perceptions Well-being Performance anxiety	High job satisfaction was reported by 90% of musicians, which was significantly higher than other occupational groups. High job satisfaction correlated with high levels of skill variety and autonomy, and with fewer conflicts in interpersonal relationships.
Kubacki, 2008	Jazz musicians	16	UK, Poland F2 M14 Age range approx. 26–65	Qualitative: In-depth biographical interview	Explore experiences of the creation of live performance	Career experiences	Organising function engagements was associated with negative affect. Participants reported both negative and positive relationships with the audience.
Mogelof and Rohrer, 2005	Symphony orchestra musicians	Survey *n =* 66 Interview *n =* 22	USA F27 M39 Age range 23–74 (mean age = 45.94)	Mixed-method case study: Cross-sectional questionnaire Interviews	Explore how musicians cope with career frustrations and disappointments	Job satisfaction Tenure Organisational status Coping behaviours	Orchestral status was an important factor relating to well-being. Elite orchestral musicians were more satisfied although job satisfaction decreased over time. Non-elite orchestral musicians were more satisfied with contribution to governance, though were less satisfied with job security and pay.
Parasuraman and Purohit, 2000	Symphony orchestra musicians	63	USA F37 M26 Age range 22–63 (mean = 33.5)	Quantitative: Cross-sectional questionnaire	Assess the effects of organisational demands on psychological health and well-being	Stressors Psychological distress Boredom stress Job dissatisfaction Job involvement Instrument group	Occupational demands of task difficulty, performance anxiety, social tension, lack of artistic integrity and work environment correlated with job dissatisfaction.
Perkins et al., 2017	Current and graduated conservatoire music students	20	UK F15 M5 Age range 18–24	Qualitative; Semi-structured interviews	Explore enablers and barriers to health and well-being in the conservatoire environment	Attitudes to health and well-being Enablers and barriers to health and well-being	Challenges to well-being included irregular schedules, time management, financial difficulties, teacher/pupil relationship, performance goals, comparison with peers, performance evaluation. Contributors to well-being included successful performance and relationships.
Quested et al., 2013	Dance conservatoire students	55	Hong Kong F41 M9 (mean age = 20.58)	Quantitative: Diary methodology	Assess relationships between autonomy support, basic psychological need satisfaction and changes in affective states across different dance situations	Dance genre Perceived autonomy support Basic psychological need satisfaction Well-being	Perceived autonomy support significantly predicted basic psychological need satisfaction. Basic psychological need satisfaction contributed to changes in affect.
Robb et al., 2018	Actors	20	Australia F10 M10 Age range 22–66	Qualitative: Semi-structured interviews	Explore factors that impact the psychological well-being of actors	Well-being Acting Demands Personal characteristics	Challenges to well-being for actors included job insecurity, financial insecurity, maladaptive alcohol consumption, perfectionistic tendencies and distressing content. Contributors to well-being included career engagement, relationships with audiences, creative expression and personal growth. Relationships with colleagues were experienced as both contributing to and detracting from well-being.
Sandgren, 2002	Opera singers	Interview *n =* 15 Survey *n =* 49	Sweden Qual: F8 M7 Age range 27–65 Quant: F25 M24 Age range 21–65	Mixed-methods: Semi-structured interviews Cross-sectional questionnaire	Explore problems, coping strategies and motivation of opera singers and how these aspects relate to mental and physical health	Demands Coping Motivational factors Somatic problems Depressive tendencies Addictive behaviour Worry Performance anxiety	Inability to sing related to negative affect. Job insecurity, rehearsal schedules and avoidance of social environments impacted on personal relationships. Performance related to positive affect and mastery.
Smith, 1989	Retired symphony orchestra musicians	14	USA M14 Age range 57–90	Qualitative: Semi-structured interview	Explore career experiences, medical problems and career perceptions	Medical problems Career	Job satisfaction related to being part of a successful team. Job dissatisfaction was related to relationships with colleagues, managing schedules and lack of recognition.

**Figure 1:**
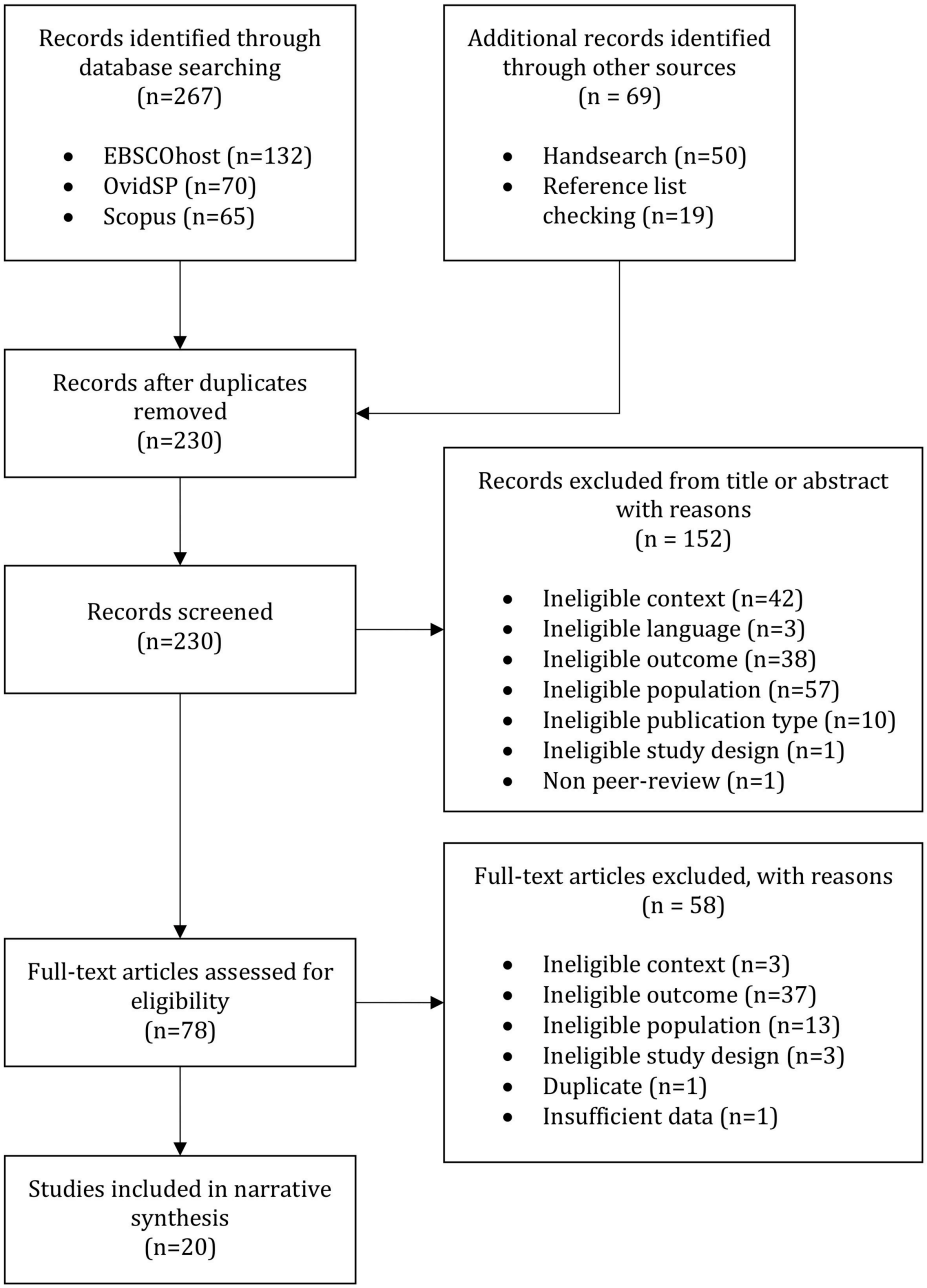
Flow diagram for study inclusion adapted from Moher et al. (2009).

Figure 1 provides details of the searches conducted and articles excluded at each stage of the review process.

### Quality Assessment

An overall quality score was assigned to each study using the MMAT scoring system (Pluye et al., 2011). Studies could be awarded a score of unclassified, 25, 50, 75 or 100%. Within this systematic review three studies were unclassified, eight studies were rated 25%, five studies were rated 50%, three studies were rated 75%, and only one study was rated 100% (Table 2). See Table 3 for details of quality assessment for each study.

**Table 2:** Total MMAT scores.

**MMAT criteria**	**No. studies**
Unclassified	3
25%	8
50%	5
75%	3
100%	1

**Table 3:** Quality assessment scores using MMAT (Pluye et al., 2011).

	**1. Qualitative**				**4. Quantitative descriptive**				**5. Mixed methods**			**Overall quality score**
**References**	**1.1**	**1.2**	**1.3**	**1.4**	**4.1**	**4.2**	**4.3**	**4.4**	**5.1**	**5.2**	**5.3**	
Abeles and Hafeli, 2014	No	Yes	No	No								^*^
Allmendinger et al., 1996	No	No	No	No	No	No	No	Can't tell	No	Yes	No	U
Ascenso et al., 2017	Yes	Yes	No	No								^**^
Bodner and Bensimon, 2008					No	No	Yes	Yes				^**^
Brodsky, 2006	Yes	Yes	Yes	No								^***^
Burgoyne et al., 1999	No	Yes	No	No								^*^
Cooper et al., 1989	No	Can't tell	No	No	No	No	No	Can't tell	No	No	No	U
Dobson, 2010b	No	No	No	No								U
Draugelis et al., 2014					Can't tell	Can't tell	Yes	Can't tell				^*^
Johansson and Theorell, 2003					Yes	Yes	No	Yes				^***^
Kenny et al., 2016a					Yes	Yes	Yes	Yes				^****^
Kivimaki and Jokinen, 1994					Yes	No	Yes	No				^**^
Kubacki, 2008	No	Yes	No	No								^*^
Mogelof and Rohrer, 2005	Can't tell	Yes	No	No	Yes	No	Yes	No	Yes	Yes	No	^*^
Parasuraman and Purohit, 2000					No	No	No	Yes				^*^
Perkins et al., 2017	Yes	Yes	No	Yes								^***^
Quested et al., 2013					Yes	No	Yes	Can't tell				^**^
Robb et al., 2018	Yes	Yes	No	No								^**^
Sandgren, 2002	Yes	Yes	No	No	No	No	No	Yes	Yes	No	No	^*^
Smith, 1989	Yes	No	No	No								^*^

* *meets 25% of MMAT criteria*.

** *meets 50% of MMAT criteria*.

*** *meets 75% of MMAT criteria*.

**** *meets 100% of MMAT criteria*.

*U, Unclassified*.

Within the included studies, sampling concerns were identified across many quantitative and qualitative papers, which failed to include reasons for non-participation in the relevant research project. Of the studies employing quantitative methodologies, six studies did not report establishing an appropriate sampling strategy to achieve statistical power and nine studies failed to ensure their sample was representative of the population under study. The validity of measures used in the research was not considered in five of the included studies using quantitative methodologies and response rates were often not reported. Considering those studies that used qualitative methodologies, all but one study failed to identify the potential influence of the researchers' epistemological perspective on the study design and the reporting of participants' experiences. Twelve of the studies using qualitative methodologies did not account for the influence of the context of data collection. In the quality assessment of the four mixed-method studies, only two studies reported an appropriate rationale for the combination of methods and no studies considered the potential limitations of the integration of different methods. This demonstrates the overall low quality of studies conducted on the relationship between occupational demands and well-being in performing artists according to the MMAT criteria.

## Summary of Studies

A total of 20 studies were included in the systematic review (Table 1). Of those, seven studies were quantitative, nine qualitative and four mixed methods. The majority of quantitative studies (*n* = 5) used cross-sectional surveys to collect data. The most frequently used method for qualitative data collection was semi-structured interviews (*n* = 7). Within the mixed-methods studies a combination of surveys and interviews were used most frequently (Cooper and Wills, 1989; Sandgren, 2002; Mogelof and Rohrer, 2005). In addition to survey and interview methods, Allmendinger et al. (1996) used observational methods and analysed archived company documents. The majority of studies were conducted with musicians (*n* = 17) and, of those, 13 studies were conducted with classical musicians. Classical musicians were situated most frequently in an orchestral context, though studies also included musicians in freelance (Dobson, 2010b), solo, chamber (Ascenso et al., 2017) and choral (Sandgren, 2002) settings. Non-classical musicians included those in jazz (Kubacki, 2008), popular (Cooper and Wills, 1989) and rock (Bodner and Bensimon, 2008) settings. Studies were also conducted with dancers (*n* = 2) and actors (*n* = 2).

Data were collected from professional performing artists in the majority of studies (*n* = 15). Only four studies included participants studying in higher education contexts at conservatoires (Quested et al., 2013; Perkins et al., 2017) and universities (Burgoyne et al., 1999; Draugelis et al., 2014). Their subjects spanned the performing arts and included music (Perkins et al., 2017), dance (Quested et al., 2013; Draugelis et al., 2014) and acting (Burgoyne et al., 1999).

Exactly half of the studies were conducted in Europe (*n* = 10) and of those, seven studies were conducted in the UK. Studies were also conducted in Sweden (Sandgren, 2002; Johansson and Theorell, 2003) and Finland (Kivimaki and Jokinen, 1994). Outside Europe, studies were conducted in the USA (*n* = 7) and Australia (*n* = 2). Further, Bodner and Bensimon (2008) collected data from participants in Israel, while data was collected in Hong Kong by Quested et al. (2013). Two studies were conducted with multi-national samples; Allmendinger et al. (1996) collected data from participants in the UK, Germany and the USA and Kubacki (2008) collected data from participants in the UK and Poland. Within the quantitative studies sample size ranged from 38 (Bodner and Bensimon, 2008) to 407 (Kenny et al., 2016a). The sample size for qualitative studies was between six (Ascenso et al., 2017) and 47 (Abeles and Hafeli, 2014).

Studies contextualised their research within a variety of conceptual frameworks, most frequently aligning with well-being (*n* = 4) or occupational stress (*n* = 3). Within those studies drawing on well-being frameworks, Robb et al. (2018) used a eudaimonic conceptualisation of well-being (Ryff, 2014), Ascenso and Perkins (2013) drew on Seligman's PERMA framework (Seligman, 2011), and Quested et al. (2013) aligned with both self-determination theory (Deci and Ryan, 1985, 2000) and basic needs theory (Deci and Ryan, 2000). Of those studies aligned with the occupational literature, a variety of concepts were considered. Johansson and Theorell (2003) discussed several models including the demand-control(-support) model (Karasek, 1979; Karasek and Thoerell, 1990), and the effort-reward imbalance model (Siegrist, 1996). Cooper and Wills (1989) drew on Seyle's model of stress (Selye, 1946) whilst Kivimaki and Jokinen (1994) cited occupational stress models by Hackman and Oldham (1976) and the job characteristics model (Fried and Ferris, 1987). Two studies utilised health conceptualisations; Perkins et al. (2017) used a health promotion framework and Sandgren (2002) took a psychosomatic perspective. A wide variety of concepts were cited in the remainder of studies; for example, Draugelis et al. (2014) used both achievement goal theory (Roberts, 2001) and self-concept theory (Vispoel, 1993, 1995).

Reflecting the diversity of conceptual approaches, a wide variety of measures was used to assess both occupational demands and well-being in those studies that used survey methods for data collection. Well-being was assessed using 16 different measures and occupational stress was represented by no fewer than five measures. Whilst some authors stated the validity and reliability of the questionnaires used, several authors used self-developed questionnaires where validity was not established (Allmendinger et al., 1996; Parasuraman and Purohit, 2000; Sandgren, 2002; Johansson and Theorell, 2003).

### Relationship Between Occupational Demands and Well-Being

The next section considers the relationship between occupational demands and well-being. In order to bring the literature together, the included studies will be discussed in light of the main occupational stress and well-being conceptualisations outlined in the introduction.

#### Job Demand-Control(-Support) Model

Several studies explored the areas of occupational demands, autonomy and social support. Cross-sectional studies suggested that occupational demands are related to well-being in symphony orchestra musicians (Kivimaki and Jokinen, 1994; Parasuraman and Purohit, 2000; Johansson and Theorell, 2003; Quested et al., 2013; Kenny et al., 2016a). Kivimaki and Jokinen (1994) found that high autonomy, high skill variety and good interpersonal relationships at work correlated with high job satisfaction in orchestral musicians. The study also found that 90% of musicians reported high job satisfaction. Multiple linear regression was used to assess the contribution of several occupational demands to well-being in a population of symphony orchestra musicians (Johansson and Theorell, 2003). Occupational demands (assessed with questions related to the quality of conductor, repertoire and rehearsals) and social support were important factors in predicting the well-being of musicians. However, contrary to the JDC model, control was not a significant factor in predicting well-being in musicians (Johansson and Theorell, 2003). Parasuraman and Purohit (2000) also assessed the contribution of occupational stressors to well-being (measured as job satisfaction), and found that lack of autonomy and low levels of social support were the biggest predictors.

Qualitative studies also reported on the dimensions of the JDC(S) model and the implications for the well-being of performing artists. Musicians discussed experiencing high levels of organisational demands, including areas such as task difficulty, heavy scheduling and time management issues, which negatively impacted on well-being (Cooper and Wills, 1989; Smith, 1989; Sandgren, 2002; Brodsky, 2006; Kubacki, 2008; Dobson, 2010b; Ascenso et al., 2017; Perkins et al., 2017). Organisation issues were discussed specifically by jazz musicians, who reported that organising function engagements could be challenging and led to the experience of negative emotions such as humiliation (Kubacki, 2008). Considering the autonomy dimension of the JDC model, orchestral musicians reported experiencing a low level of independence, which was related to lower levels of well-being in terms of job satisfaction and negative affect (Brodsky, 2006; Dobson, 2010b). However, jazz musicians and those working in freelance settings discussed opportunities to input into creative and management decisions as positive contributors to well-being (Cooper and Wills, 1989; Dobson, 2010b). Performing artists reflected on the importance of social support for their well-being, identifying the positive benefits of working alongside individuals with a shared interest (Smith, 1989; Robb et al., 2018).

#### Effort-Reward Imbalance Model

Extrinsic factors of the reward dimensions of the ERI model (remuneration, job security, career opportunities and esteem) are particularly relevant to performing artists (Cooper and Wills, 1989; Allmendinger et al., 1996; Sandgren, 2002; Mogelof and Rohrer, 2005; Brodsky, 2006; Abeles and Hafeli, 2014; Kenny et al., 2016a; Ascenso et al., 2017; Robb et al., 2018). Questionnaire research suggests that performing artists reported low satisfaction with remuneration (Allmendinger et al., 1996; Kenny et al., 2016a), however qualitative reports recognised that some performing artists were satisfied with being remunerated for a job they valued (Brodsky, 2006). Performing artists also reported low satisfaction with job security (Robb et al., 2018). Further, the lack of job security was related to experiencing financial issues and actors reported not being able to achieve financial milestones relevant to their age (Robb et al., 2018). Symphony orchestra musicians discussed limited opportunities for career progression, which was related to lower job satisfaction (Brodsky, 2006). However, qualitative data from musicians working in a regional orchestra found that participants appreciated opportunities to contribute to governance decisions (Mogelof and Rohrer, 2005). This was reported alongside quantitative data from the same participants who found their job satisfaction increased over time. The lack of opportunities for career progression within orchestral careers may have led to musicians seeking out opportunities to develop new skills outside their principal role in areas such as teaching curriculum-based music lessons (Abeles and Hafeli, 2014), learning a new instrument and familial responsibilities (Ascenso et al., 2017).

The ERI model also considers the impact of the intrinsic factor of over-commitment. This may be particularly relevant to actors, who reported a high level of identification and passion for their careers (Burgoyne et al., 1999; Robb et al., 2018). While these high levels of commitment could contribute positively to well-being for some individuals, the reported levels of identification had a negative impact on well-being outcomes during difficult times, such as periods of unemployment (Robb et al., 2018). Further complexity regarding identity was experienced by actors, who discussed the blurred boundaries between their personal identity and that of the characters they portrayed (Burgoyne et al., 1999; Robb et al., 2018). Immersion in the role being performed meant individuals were unable to re-establish their own personalities following performances and led to some actors losing control on-stage with incidents of unintended violence (Burgoyne et al., 1999).

#### Job Demands-Resources Model

Inclusion of the most salient occupational demands, as suggested in the JD-R model allows for a wider exploration of the demands that musicians face. Whilst the positive impact of social support on well-being is discussed above, the frequent need to work as part of a large team exposes performing artists to interpersonal demands, which may have a negative impact on well-being (Cooper and Wills, 1989; Allmendinger et al., 1996; Burgoyne et al., 1999; Parasuraman and Purohit, 2000; Mogelof and Rohrer, 2005; Kenny et al., 2016a; Ascenso et al., 2017; Robb et al., 2018). Performing artists reported low satisfaction with management (Allmendinger et al., 1996), with musicians working in pits reporting lower satisfaction than those working in stage orchestras (Kenny et al., 2016a). Mogelof and Rohrer (2005) suggested that satisfaction with management was related to the status of the orchestra and musicians employed by lower status orchestras reported significantly less satisfaction with management. Satisfaction with management was also explored qualitatively (Burgoyne et al., 1999; Ascenso et al., 2017). Musicians highlighted tension between their goals as musicians and those of management (Ascenso et al., 2017), whilst actors discussed the negative impact that a director's working style could have on well-being outcomes (Burgoyne et al., 1999).

Interpersonal relationships with colleagues also had the potential to impact negatively on the well-being of performing artists (Cooper and Wills, 1989; Smith, 1989; Kenny et al., 2016a; Ascenso et al., 2017; Robb et al., 2018). This was through experiences of performing in groups that were mixed in technical ability (Cooper and Wills, 1989; Smith, 1989), incidents of bullying (Kenny et al., 2016a; Robb et al., 2018), temporary relationships with colleagues due to transient organisational affiliation (Robb et al., 2018) and competition amongst peers (Perkins et al., 2017).

Bakker and Demerouti (2007) suggested that both emotional demands and performance feedback could be considered in models of occupational stress. The nature of performing artists' roles requires individuals to portray a wide range of emotions through expressive mediums. Actors reported that performing scenes of a traumatic nature was associated with low well-being (Burgoyne et al., 1999; Robb et al., 2018) and actors reported imaging distressing personal situations to emotionally connect with characters, which had a negative impact on well-being (Burgoyne et al., 1999). Due to the public nature of performance settings, performing artists recognised that they were open to external criticism (Cooper and Wills, 1989; Smith, 1989; Sandgren, 2002; Brodsky, 2006; Ascenso et al., 2017; Perkins et al., 2017). Performance feedback perceived as criticism had a negative impact on performing artists well-being (Sandgren, 2002) and a perceived lack of recognition for their work was related to lower job satisfaction for orchestral musicians (Smith, 1989).

The inclusion of resources in the JD-R model is also advantageous when assessing the relationship between occupational demands and well-being of performing artists. Resources may buffer the impact of occupational demands on well-being and facilitate personal development (Schaufeli and Bakker, 2004). Whilst it was not the intention of this systematic review to provide a comprehensive report of occupational resources and their impact on well-being, pertinent findings from the included studies are discussed. Musicians viewed music-making as an activity that they enjoyed in and of itself, which positively impacted on well-being by increasing job satisfaction (Brodsky, 2006; Ascenso et al., 2017). Further, making music in a performance setting contributed positively to well-being and positive performance experiences were related to positive affective outcomes and satisfaction (Sandgren, 2002; Ascenso et al., 2017; Perkins et al., 2017). Performance was related to the experience of heightened emotional responses for musicians and participants discussed experiencing “peak” performance states (Brodsky, 2006; Ascenso et al., 2017; Perkins et al., 2017). However, whilst these heightened affective states were perceived positively by participants, they were also seen as a challenge to the well-being of some musicians, who experienced difficulty in regulating their emotions following performances (Brodsky, 2006; Bodner and Bensimon, 2008).

Social aspects of performing artists' occupations may also be considered as resources. In addition to the role of social support of colleagues, interpersonal relationships with audiences contributed positively to well-being. Sharing performances with the audience was seen to contribute to positive affect (Sandgren, 2002; Brodsky, 2006; Robb et al., 2018). Additionally, the role of task-climate may be viewed as a resource, which forms part of the social environment of performing artists and has the potential to impact on well-being (Draugelis et al., 2014). A task-climate is present when individuals are rewarded for personal effort, perceive errors as opportunities for improvement and participate in learning decisions (Ntoumanis and Biddle, 1999). The perception of the climate may be influenced by those in leadership positions, such as managers and teachers, and others within the social environment. In a study with dancers, Draugelis et al. (2014) suggested that positive perceptions of task-climate were an important factor in predicting well-being with higher perceptions of task-climate associated with higher well-being. In a separate study with dancers, Quested et al. (2013) assessed the relationship between the social environment and well-being. The authors examined the concept of relatedness, which is a construct taken from self-determination theory (Deci and Ryan, 2000), and found that it was an important predictor of well-being.

## Discussion

### Summary of Main Findings

A total of 20 articles met the inclusion criteria for the systematic review, of which only four studies met the MMAT quality assessment score of 75% or above. This suggests a lack of high quality research in this area meaning the synthesis of results for this systematic review should be interpreted with caution. Classical musicians were the most frequent participants, although there is scope within this field to conduct further high-quality research. Only two studies were conducted with dancers (Quested et al., 2013; Draugelis et al., 2014) and a further two with actors (Burgoyne et al., 1999; Robb et al., 2018). No studies were identified for this systematic review that assessed the occupational demands and well-being in either circus artists or comedians. A wide variety of conceptual frameworks from occupational stress and well-being were used and this was reflected in the range of measures. Whilst theoretical frameworks were considered, few quantitative studies included measures that were aligned with those frameworks (Kivimaki and Jokinen, 1994; Quested et al., 2013; Draugelis et al., 2014). The lack of a firm theoretical basis in many studies, precludes the testing of appropriate models of occupational stress and well-being. Additionally, the use of measures that did not have established validity or reliability and sampling issues hampers the progress that can be made in this field. Furthermore, the lack of identification of epistemological beliefs from qualitative researchers is an issue for research in this field.

The studies presented in this review suggest that several frameworks of occupational stress and well-being may be appropriate for exploring the relationship between occupational demands and well-being in performing artists. Studies conducted with musicians, suggest that the JDC(S) model may be suitable as organisational demands, autonomy and social support were all seen to contribute to well-being (Kivimaki and Jokinen, 1994; Parasuraman and Purohit, 2000; Johansson and Theorell, 2003). The importance of these areas for the well-being of performing artists was confirmed in qualitative research reports (e.g., Kubacki, 2008; Dobson, 2010b; Perkins et al., 2017). Considering the ERI model, performing artists reported low levels of occupational rewards in the form of remuneration, job security and career progression (e.g., Allmendinger et al., 1996; Mogelof and Rohrer, 2005; Abeles and Hafeli, 2014). Taking this into account alongside the high number of occupational demands described above, this leaves performing artists vulnerable to experiencing occupational stress due to the imbalance between effort and reward. Furthermore, the ERI model suggests that over-commitment may play a role in the experience of occupational stress. Actors, in particular, displayed a significant commitment to their work (Burgoyne et al., 1999; Robb et al., 2018), which may contribute to the imbalance of high effort and low reward.

However, the JDC(S) and ERI models do not take into account all the occupational demands experienced by performing artists. Conceptualisations of stress that allow for a broader inclusion of occupational demands may be better suited to exploring the working environment of performing artists, due to the inclusion of areas such as interpersonal demands, emotional demands and performance feedback. The requirement to work with large groups of people and respond effectively to interpersonal cues means that performing artists are exposed to high levels of interpersonal demands (e.g., Cooper and Wills, 1989; Burgoyne et al., 1999; Parasuraman and Purohit, 2000; Kenny et al., 2016a). Emotional demands and performance feedback are also inherent within performing arts careers and were discussed frequently in qualitative reports (e.g., Sandgren, 2002; Brodsky, 2006; Ascenso et al., 2017).

It is also necessary to consider the most applicable conceptualisation of well-being for performing artists. Performing artists reported well-being outcomes related to a hedonic conceptualisation of well-being and explored affective and cognitive well-being outcomes. Job satisfaction (Kivimaki and Jokinen, 1994; Parasuraman and Purohit, 2000) and satisfaction with specific aspects of performing arts careers [e.g., pay, job security; (Allmendinger et al., 1996; Kenny et al., 2016a)] were assessed and meaningful data were reported. Additionally, both positive and negative affective responses were discussed in relation to performing. In particular, musicians reported experiencing guilt as a result of making mistakes during performance (Sandgren, 2002; Dobson, 2010b; Perkins et al., 2017). Jazz musicians also reported feeling shame when organising their own function engagements (Kubacki, 2008). An eudaimonic conceptualisation of well-being may also be a suitable perspective to view the well-being of performing artists. Both Robb et al. (2018) and Ascenso et al. (2017) used a eudaimonic framework to guide qualitative explorations of well-being of performing artists. Specifically, performing artists highlighted the meaning they derived from their careers and their commitment to their chosen art form (Burgoyne et al., 1999; Ascenso et al., 2017; Robb et al., 2018) This framework seems relevant for understanding the well-being experiences of performing artists and quantitative research using this framework would illuminate the applicability of this construct.

### Limitations

Various limitations exist within this field. One limitation within the studies included in this systematic review is the sensitive nature of the topic and potential unwillingness of participants to openly report their experiences of occupational stress and well-being, particularly in an environment where they experience a lack of perceived or actual job security. A study with orchestral musicians suggested that organisational norms led musicians to conceal health issues due to the potential repercussions on relationships with colleagues and management (Rickert et al., 2014). Further, the potential influence of external factors (e.g., socioeconomic, health, lifestyle) are not systematically explored in the included studies and may also impact on the well-being of performing artists. Socioeconomic factors and coping behaviours may moderate the relationship between occupational demands and well-being (Vaag et al., 2014).

The lack of theoretically informed study designs, as highlighted in the synthesis of this systematic review, is a significant issue in this field. Furthermore, few studies have been conducted using contemporary conceptualisations of occupational stress and well-being such as the Demands Resources and Individual Effects model (DRIVE; Mark and Smith, 2008) or eudaimonic well-being (Ryff, 2014). The absence of theory-driven research limits the progress that can be made in this field and the development of evidence-based interventions.

One limitation of this systematic review concerns the potential for incomplete retrieval of studies on the topic due to the restriction of the search to articles published in the English language (Grégoire et al., 1995). This decision was taken due to the availability of resources for the study. Study publication bias and outcome bias are also potential limitations of individual studies in this systematic review (Dwan et al., 2013). The low MMAT scores attributed to the majority of studies means that the findings of this review should be interpreted with caution. Specifically, the issues with sampling across studies identified in the MMAT limit the generalisability of the quantitative findings. In terms of qualitative research findings, the lack of acknowledgement of researchers' epistemological views is an issue for the current evidence base, due to the potential to impact on the interpretation of research findings.

### Conclusions

#### Implications for Future Research

This systematic review highlights the paucity of high quality research that has been conducted on the relationship between occupational demands and well-being in performing artists. Further exploration of this issue from both quantitative and qualitative perspectives would enhance our knowledge of this field and the following observations are made to guide future research foci. Firstly, the wide variety of conceptual frameworks of both occupational stress and well-being in the included studies highlights the lack of agreement in the literature; an issue previously explored by Dodge et al. (2012). A holistic approach, which considers occupational demands, mediating factors and well-being outcomes could be achieved through adopting contemporary approaches to researching occupational stress. This would allow for a greater understanding of the impact of occupational demands on well-being whilst facilitating accuracy and consistency within the literature.

Secondly, future research should seek to employ methodologically robust study designs. This should encompass the use of sampling procedures, which justify the choice of participants and use power calculations to ensure adequate numbers of participants are recruited for statistical tests. Further, the choice of measures needs more in-depth consideration. The included studies in this systematic review used a wide variety of measures, which in some cases did not align with the theoretical positioning of the research. Measures should align with the relevant theoretical frameworks and demonstrate reliability and validity in order to provide meaningful results. Due to the lack of measures available specifically for musicians, several authors developed their own questionnaires. However, these have not been subject to rigorous testing to demonstrate adequate validity. Future research should seek to provide a questionnaire which is specific to the occupational demands that musicians experience. Using such a measure would allow comparisons across research studies and progress understanding in this field. One avenue for such exploration is the Psychological Risks Questionnaire for Musicians developed by Jacukowicz and Wezyk (2018).

Thirdly, using a broader range of study designs would enable developments in this field. The majority of quantitative studies carried out on this topic have been cross-sectional, meaning that causation cannot be implied. Longitudinal studies are needed to allow a greater exploration of the causal effects of occupational stress on well-being over longer periods of time. The study by Mogelof and Rohrer (2005) considered in this review suggested that satisfaction decreased over time for those in elite orchestras, but increased over time for those in regional orchestras. Further research is necessary to explore this issue. A considered approach to study design will allow for the use of more advanced statistical tests, such as structural equation modelling and path analysis. This will provide a multivariate perspective of well-being in performing artists. Systematically exploring the contribution of occupational stress variables to well-being will also illuminate the differential effects of individual experiences. Further, study designs with control groups that develop evidence-based interventions are needed for this population in order to facilitate the development of resources to cope with the occupational demands inherent in performing arts careers.

Considering the whole stress process including the role of resources and appraisals will aid developments in this field. Few studies in this systematic review considered the potential role resources may have on the relationship between occupational demands and well-being. Transactional models of stress, such as the cognitive-motivational-relational model (Lazarus, 1999), suggest that the relationship between occupational demands and well-being may be affected by an individual's appraisal of occupational demands. To date, no studies have explored the effect of appraisal on the relationship between demands and resources in performing artists. Future research should extend the understanding of the role of coping on the relationship between occupational demands and well-being. Such research would help to illuminate the reasons for inter- and intra-individual differences in well-being outcomes when similar occupational demands are present. Exploring the effectiveness and impact of coping strategies on well-being would facilitate the development of evidence-based interventions for this population.

Whilst the quality assessment conducted using the MMAT suggests that much of the research in this area is not of high quality, a significant issue was the reporting standards of many studies. Researchers should follow reporting guidelines to ensure the completeness of the dissemination of research findings. This is important both for transparency and the production of high quality research that offers more accurate insights.

#### Conclusion

This systematic review highlights the need for more high quality research on the relationship between occupational stress and well-being. One of the main findings of this review was the lack of theoretical basis for work conducted in this area and the resulting asynchronism of measures. Frameworks which offer a holistic perspective of the relationship between occupational demands and well-being, such as the DRIVE model (Mark and Smith, 2008), may be appropriate for exploring the relationship between occupational stress and well-being. Further consideration of the role of appraisal would add greater depth to the understanding of the occupational stress process in performing artists. In terms of well-being, both hedonic and eudaimonic perspectives of well-being are relevant to musicians.

Performing artists are exposed to a range of organisational, social and emotional demands, which impact negatively on well-being. These include touring, scheduling, interpersonal relationships with colleagues, performance, and feedback. They also face low rewards, in the form of remuneration, job security and opportunities for career progression. Resources such as music-making, performance and interactions with the audience had a positive impact on the well-being of performing artists. Further exploration of the stress and well-being process will facilitate the understanding of occupational demands and well-being within this population and assist with the development of evidence-based interventions for performing artists. Such interventions could include involvement in education programmes (Abeles and Hafeli, 2014), community engagement (Preti and Welch, 2013; Ascenso, 2016) and chamber music performances (Parasuraman and Purohit, 2000). This would allow performing artists to acquire appropriate skills to cope with the inevitable occupational demands they face and to continue working in careers they remain passionate about.

## Author Contributions

All authors contributed to the conception, design, and analysis of the study. SW was primarily responsible for data collection and interpretation. SW drafted the initial manuscript and all authors contributed to manuscript revisions, read, and approved of the submitted version.

### Conflict of Interest Statement

The authors declare that the research was conducted in the absence of any commercial or financial relationships that could be construed as a potential conflict of interest.
