# Supplementary material for: The Relationship Between Occupational Demands and Well-Being of Performing Artists: A Systematic Review
Source: Front Psychol. 2019 Mar 4;10:393. doi: 10.3389/fpsyg.2019.00393 (PMC6409325; doi:10.3389/fpsyg.2019.00393)
Supplement: Supplementary file 1 [file Data_Sheet_1.docx]

**Appendix A**

**Criteria for inclusion and exclusion of studies**

|  | | Inclusion Criteria | Exclusion criteria |
| --- | --- | --- | --- |
| Population | Occupation | - Professional OR student performing artists - Any one of the following performing artist categories: musicians, dancers, actors, circus artists, comedians, conductors - Individuals studying performing arts awards in the following educational settings: conservatoires, colleges, training institutions, higher education institutions, specialist performing arts training institutions | - Those not working as performing artists OR those not studying for an award in the field of the performing arts - Authors, journalists, writers, sculptors, painters, composers, conductors, choreographers, film directors, stage directors, photographers, image and sound recording personnel |
|  | Age | - Adults aged 18 and above | - Children and young people aged 17 and below |
| Comparator | | - Performing artists, non-performing artists, no comparator specified |  |
| Outcomes | | - Assesses relationship between occupational demands and well-being   Occupational demands operationalised as “physical, social, or organisational aspects of the occupation that require sustained physical or mental effort” (Demerouti et al., 2001, 501).  Well-being operationalised as psychological functioning of the individual represented by only cognitive evaluations relating to the quality of life, or cognitive evaluations and affective outcomes combined relating to the quality of life.  Cognitive evaluations include life satisfaction, job satisfaction, environmental mastery, autonomy, self-acceptance, relations with others, purpose in life, personal growth. Affective evaluations include positive affect and negative affect (Diener et al., 1999; Ryff, 2014). | - Measures only one outcome from occupational demands or well-being - Does not assess the relationship between occupational demands and well-being   Studies measuring only affective well-being outcomes (i.e. positive or negative affect) and not cognitive evaluations. |
| Study Design | | - Qualitative (all study designs) - Quantitative (all study designs) - Mixed-methods (all study designs) | - Systematic review - Literature review - Commentary |
| Publication Type | | - Peer-reviewed journal articles | - Non peer-reviewed articles - Editorials and forewords - Books and book chapters - Book reviews - Book synopses - Conference proceedings - Abstracts and unpublished theses |
| Language | | - English language | - Non-English language |
| Date | | - No date filter - up to date searched (13/10/17) |  |

**Appendix B**

**Data Extraction Form**

| **Main Author (Year)** |  | | | |
| --- | --- | --- | --- | --- |
| **Full Reference** |  | | | |
| **Journal Impact Factor** |  | | | |
| **Reason for inclusion in systematic review** | | | | |
| **Inclusion criteria** | | | | **Delete/Describe how meets inclusion criteria** |
| **Peer-reviewed journal** | | | | Yes/No |
| **English language** | | | | Yes/No |
| **Professional performing artists OR students** | | | | Professional performing artists/Students |
| **Aged 18+** | | | | Yes/No |
| **Measures Occupational Demands (give details):** | | | |  |
| **Measures well-being (give details):** | | | |  |
| **Assess the relationship between occupational demands and well-being (give details):** | | | |  |
| **Study Characteristics** | | | | |
| **Participant characteristics** | **Age** |  | | |
|  | **Gender** |  | | |
|  | **Occupation** |  | | |
|  | **Other** |  | | |
| **Context** | (e.g. symphony orchestra, conservatoire) | | | |
| **Study location/ country** |  | | | |
| **Sampling method** |  | | | |
| **Response rate** |  | | | |
| **Aims/ Research question** |  | | | |
| **Study design** |  | | | |
| **Conceptual framework** |  | | | |
| **Method** | | | | |
| **Variables/ Themes explored** |  | | | |
| **Outcome measures** | **Occupational stress measures** | |  | |
|  | **Well-being measures** | |  | |
| **Validity OR Credibility** | (i.e. were the measures used valid, is credibility discussed e.g. triangulation of analysis) | | | |
| **Method of analysis** |  | | | |
| **Results summary** | | | | |
| **Results summary** |  | | | |
|  | Findings specific to occupational demands | | | |
|  | Findings specific to well-being domains (e.g. positive affect, negative affect, life satisfaction, job satisfaction, environmental mastery, autonomy, self-acceptance, purpose in life, relationships with others, personal growth) | | | |
| **Author identified limitations** |  | | | |
| **Additional limitations** |  | | | |
| **Future research direction** |  | | | |
| **Funding Body/Sponsor** |  | | | |
| **Notes** | | | | |
|  | | | | |

**Appendix C**

**Articles Excluded at Full-text**

| Main Author | Date | Journal | Reason for Exclusion |
| --- | --- | --- | --- |
| Adams-Price et al. | 2007 | Int J of Aging Hum Dev | Wrong population: Participants are not professional jewellery-makers; recruited from discussion group. Does not mention employment status. |
| Bille et al. | 2013 | Econ Lett | Wrong population: includes authors, journalists, writers, Sculptors, painters, Composers, Choreographers, directors, Photographers, image and sound recording equipment operators, Clowns, magicians |
| Boerner et al. | 2007 | Psychol Music | Wrong outcome: Does not measure well-being as operationalised in for this systematic review |
| Bos | 2010 | J Sing | Wrong outcome: Does not measure well-being |
| Bradshaw et al. | 2005 | Consump Mark Cult | Wrong outcome: Does not measure well-being |
| Brandfonbrener | 1986 | Med Probl Perform Ar | Wrong outcome: Does not measure well-being. Wrong population: participants under 18 years of age |
| Brandfonbrener | 1988 | Med Probl Perform Ar | Wrong outcome: Does not measure well-being |
| Brandfonbrener | 2000 | Med Probl Perform Ar | Wrong outcome: Does not measure well-being |
| Brandfonbrener | 2005 | Med Probl Perform Ar | Wrong outcome: Does not assess the relationship between occupational demands and well-being |
| Brandfonbrener | 1989 | Med Probl Perform Ar | Wrong outcome: Does not assess the relationship between occupational demands and well-being |
| Brandfonbrener | 2005 | Med Probl Perform Ar | Article is a repeat of two articles that have already been excluded after reading at full-text |
| Cahalan et al. | 2013 | J Dance Med Sci | Wrong outcome: Does not assess the relationship between occupational demands and well-being |
| Cupido | 2016 | Muziki | Wrong outcome: Does not assess the relationship between occupational demands and well-being |
| Demirbatir et al. | 2013 | Sci Res | Wrong outcome: Does not measure occupational demands |
| Dobson et al. | 2015 | Psychol Music | Wrong outcome: Does not measure well-being |
| Evans | 2003 | Med Probl Perform Ar | Wrong outcome: Does not measure well-being |
| Gabriel | 1977 | Psychol Music | Wrong outcome: Does not measure occupational demands; Wrong context: Emotional responses to music. |
| Greben | 1999 | Med Probl Perform Ar | Wrong outcome: Does not measure well-being; Wrong study design: Commentary article. Observations from working with performing artists. |
| Guzmán et al. | 2014 | Rev int med Cienc act fis deporte | Wrong population: participants under 18 years of age |
| Hamilton et al. | 1994 | Med Probl Perform Ar | Wrong outcome: Does not measure well-being |
| Hancox et al. | 2017 | Pers Indiv Differ | Wrong population: participants includes children and those under 18 years: Mean age =15.57 |
| Haslam, et al. | 2009 | Stress Health | Wrong outcome: Does not assess the relationship between occupational demands and well-being |
| Heath | 2004 | Nurs Older People | Wrong outcome: Does not assess occupational demands. Context: Older people |
| Hernandez et al. | 2009 | Med Probl Perform Ar | Wrong outcome: Does not measure well-being |
| Holst et al. | 2012 | Int Arch Occ Env Hea | Wrong outcome: Does not assess the relationship between occupational demands and well-being |
| Huddy | 2016 | Perf Enhancement Health | Wrong population: Age: 17-22; Wrong outcome: Does not measure well-being; Does not measure occupational demands |
| Jenkins et al. | 2014 | J Tour Cult Change | Wrong context: examines the impact of the tourism industry from a socio-political perspective on artists. Focuses on economic well-being. Focuses on the socio-political environment rather than specific occupational demands related to artists |
| Jeong et al. | 2017 | Sustainability | Wrong outcome: Does not measure occupational demands, but perceived "gap in the work conditions". Defined as the gap between expected work conditions and actual work conditions |
| Kenny et al. | 2014 | Psychol Music | Wrong outcome: Does not measure well-being according to operationalisation for this systematic review. Does not measure occupational demands |
| Lamont | 2012 | Psychol Music | Wrong population: includes musicians studying psychology, which is not a performing arts award |
| Lee et al. | 2015 | Occup Med | Wrong outcome: Does not measure occupational demands. Measures injury, which is not an occupational demand as operationalised for this review. Injury may be considered an outcome of the physical demands of playing an instrument. |
| Liburd et al. | 2009 | Tour Hosp Res | Wrong context: Context is within an abnormal occupational setting of a festival. Not the regular context of those involved |
| Manturzewska | 1978 | Psychol Music | Wrong outcome: Does not assess occupational demands or well-being. |
| Maxfield | 2015 | J Sing | Wrong study design: Critical review |
| Maxwell | 2015 | About Perf | Wrong population: participants under 18 years old. |
| Meltzer | 2004 | J Occup Sci | Wrong outcome: Does not measure well-being. |
| Mundet-Boloset al. | 2017 | Rev Cercet Inter Soc | Wrong study design: Review. Wrong population: not professional musicians |
| No author | 1980 | Am J Occup Ther | Wrong outcome: Does not measure occupational demands. Wrong study design: Commentary |
| No author | 1987 | Am J Occup Ther | Wrong study design: information announcements |
| Quested et al. | 2009 | J Dance Med Sci | Insufficient information on population: Does not state that participants are professional or studying for performing arts awards in educational setting |
| Quested et al. | 2011 | J Sport Exercise Psy | Wrong outcome: Does not assess the relationship between occupational demands and well-being |
| Quested et al. | 2011 | Psychol Sport Exerc | Wrong population: participants under 18 years old |
| Quested et al. | 2010 | J Sport Exercise Psy | Wrong population: participants under 18 years old |
| Raeburn | 1987 | Med Probl Perform Ar | Wrong outcome: Does not assess the relationship between occupational demands and well-being |
| Raeburn | 1987 | Med Probl Perform Ar | Wrong outcome: Does not assess the relationship between occupational demands and well-being |
| Runco | 1995 | Empir Studies Arts | Wrong population: Participants are not performing artists |
| Sanal et al. | 2014 | Psychol Music | Wrong outcome: Well-being is not operationalised as for this systematic review |
| Schmalenberger et al. | 2009 | J Am Geriatr Soc | Wrong outcome: Does not measure occupational demands. Assesses the impact of breast cancer therapy and rehabilitation |
| Singha et al. | 2016 | J of Psychosoc Res | Wrong outcome: Does not measure occupational demands |
| Snooks | 1984 | J Aust Stud | Wrong context: income of Australian Artists. Does not mention occupational demands or well-being in full text |
| Steiner et al. | 2013 | J Cult Econ | Wrong outcome: Does not assess the relationship between occupational demands and well-being, due to insufficient population size |
| Stenberg | 2016 | Int J Qual Stud Health | Wrong outcome: Does not assess the well-being of artists |
| Stewart et al. | 2016 | Psychol Music | Wrong population: Participants are amateur choral and solo singers |
| Tuisku | 2016 | Med Probl Perfrom Ar | Wrong population: participants under 18 years old |
| van Staden et al. | 2009 | J Dance Med Sci | Wrong outcome: Does not measure well-being |
| Walker et al. | 2017 | J Sing | Wrong population: Applied psychology for music teachers. Wrong outcome: Does not measure the relationship between occupational demands and well-being |
| Westby | 1960 | Soc Forces | Wrong outcome: Does not measure well-being |
| Wills et al. | 1987 | Stress Med | Wrong outcome: Does not measure occupational demands |
